# Supplementary material for: Gene interaction network analysis in multiple myeloma detects complex immune dysregulation associated with shorter survival
Source: Blood Cancer J. 2023 Nov 30;13(1):175. doi: 10.1038/s41408-023-00935-2 (PMC10687027; doi:10.1038/s41408-023-00935-2)
Supplement: Supplementary file 1 — Supplementary tables and figures [file 41408_2023_935_MOESM1_ESM.docx]

**Supplementary Figure 1. Average silhouette score according to number of clusters.**

The average silhouette score for hierarchical clustering based on (A) copy number aberration and (B) RNA-sequencing defined the optimal number of clusters to be 8 for copy number and 6 for RNA-sequencing.

A B


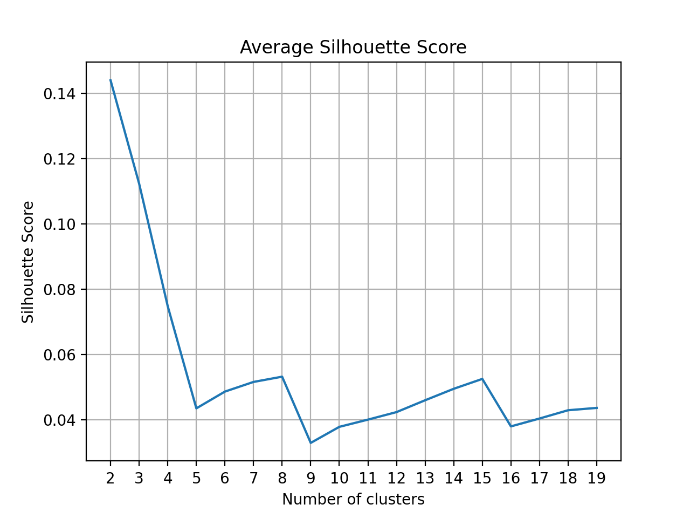

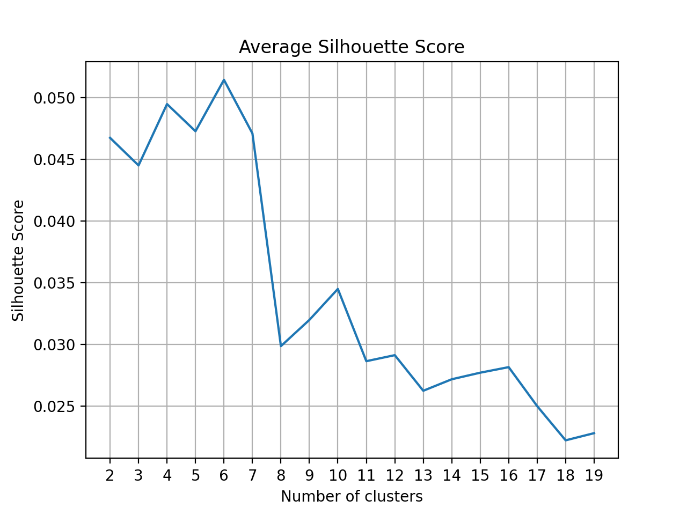


**Supplementary Figure 2. Heatmap showing the overlap between subjects in the RNA based clustering results and CNA based clustering results.**

**
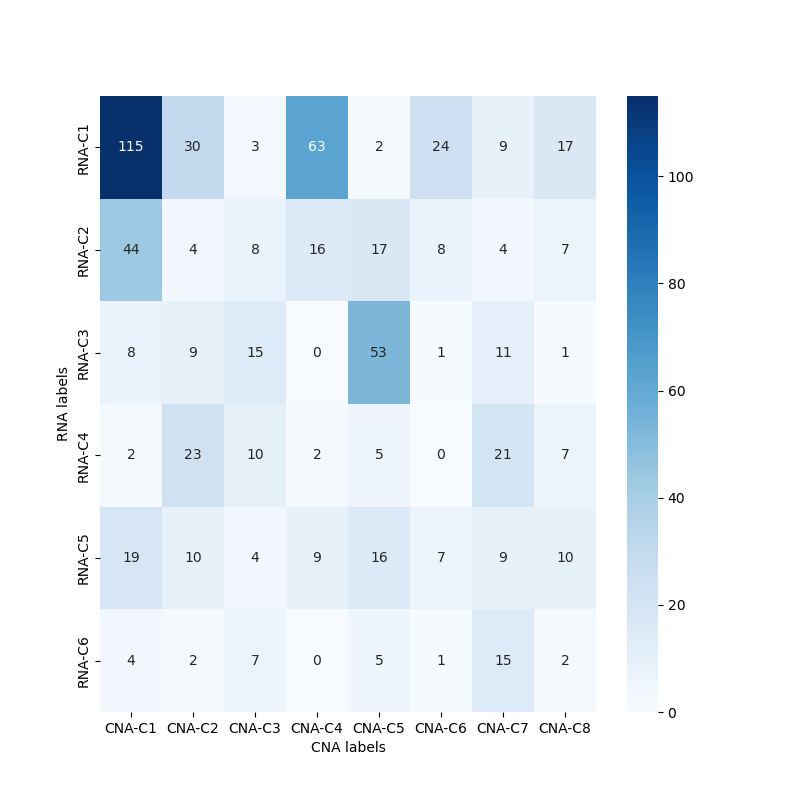
**

**Supplementary Figure 3. Heatmaps showing the relationship between common markers of MM in the study’s sample.** Top) Heatmap visualizing the CNA results presented in Supplementary Table 2. Each value in a cell represents the fraction of subjects within a cluster that are positive for a given MM marker. Bottom) Heatmap visualizing the presence of a MM marker by subject, delineated by cluster number.


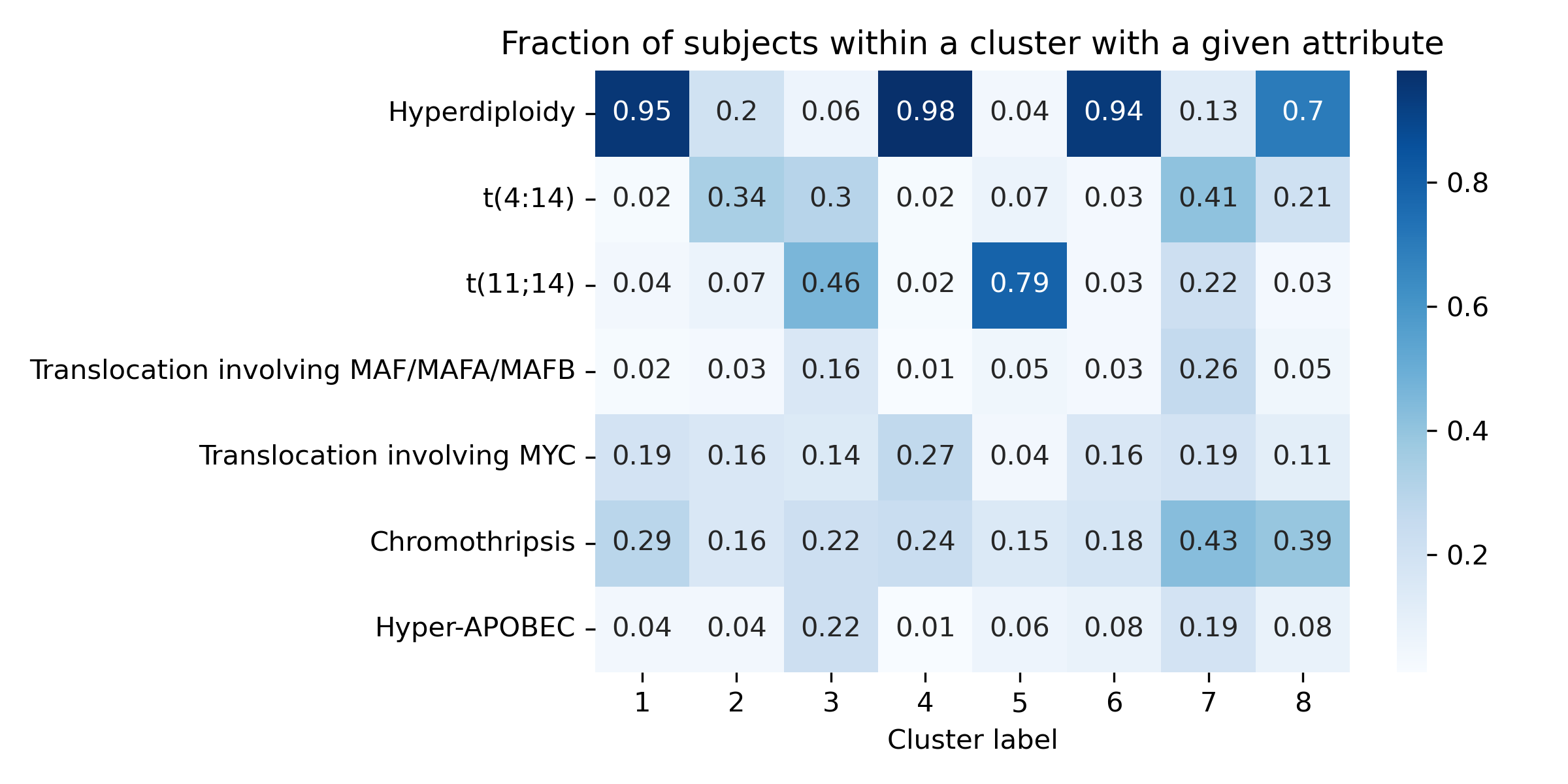


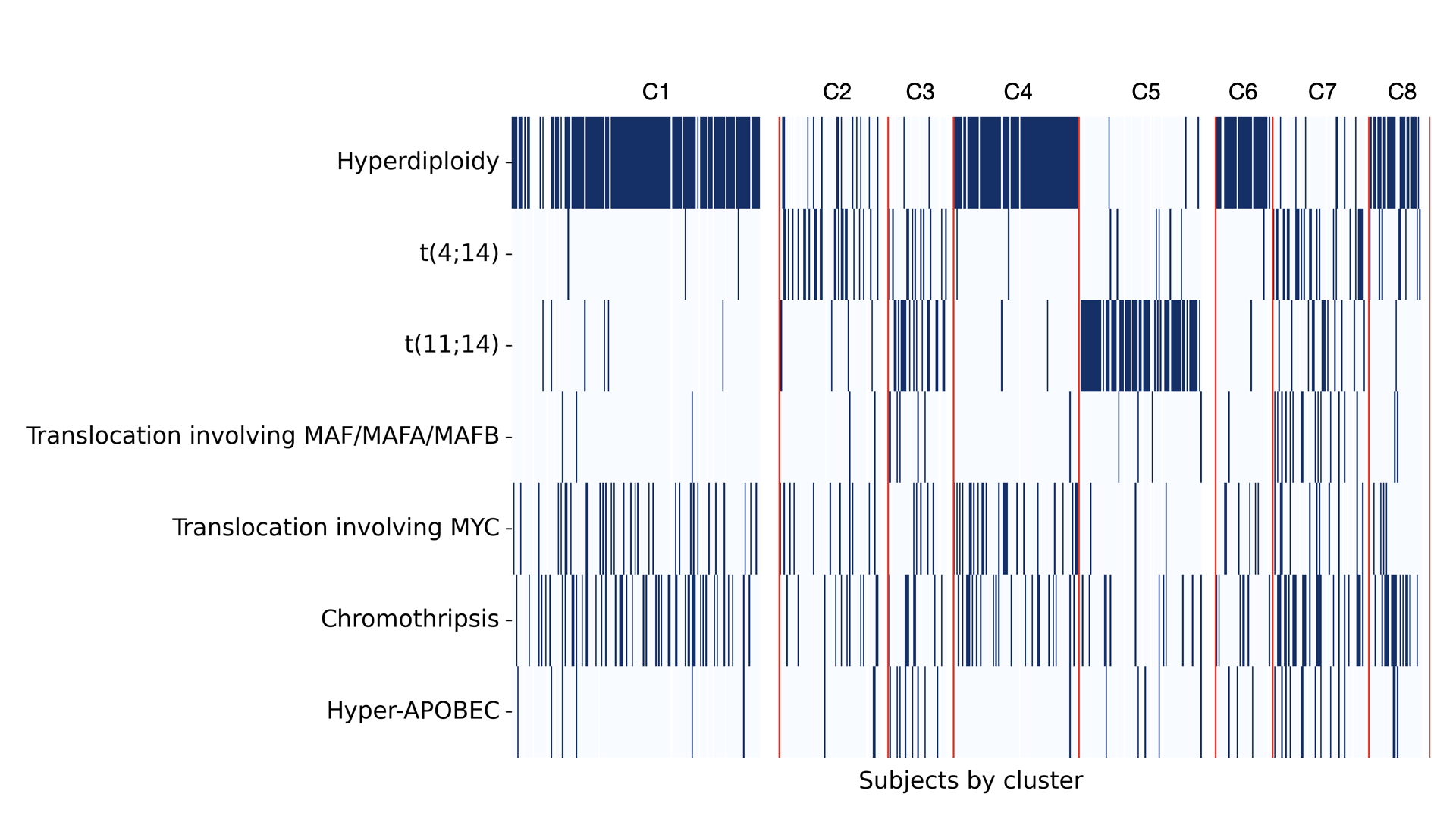


**Supplementary Figure 4. Heatmaps showing the relationship between common markers of MM in the study’s sample.** Top) Heatmap visualizing the RNA-seq results presented in Supplementary Table 3. Each value in a cell represents the fraction of subjects within a cluster that are positive for a given MM marker. Bottom) Heatmap visualizing the presence of a MM marker by subject, delineated by cluster number.


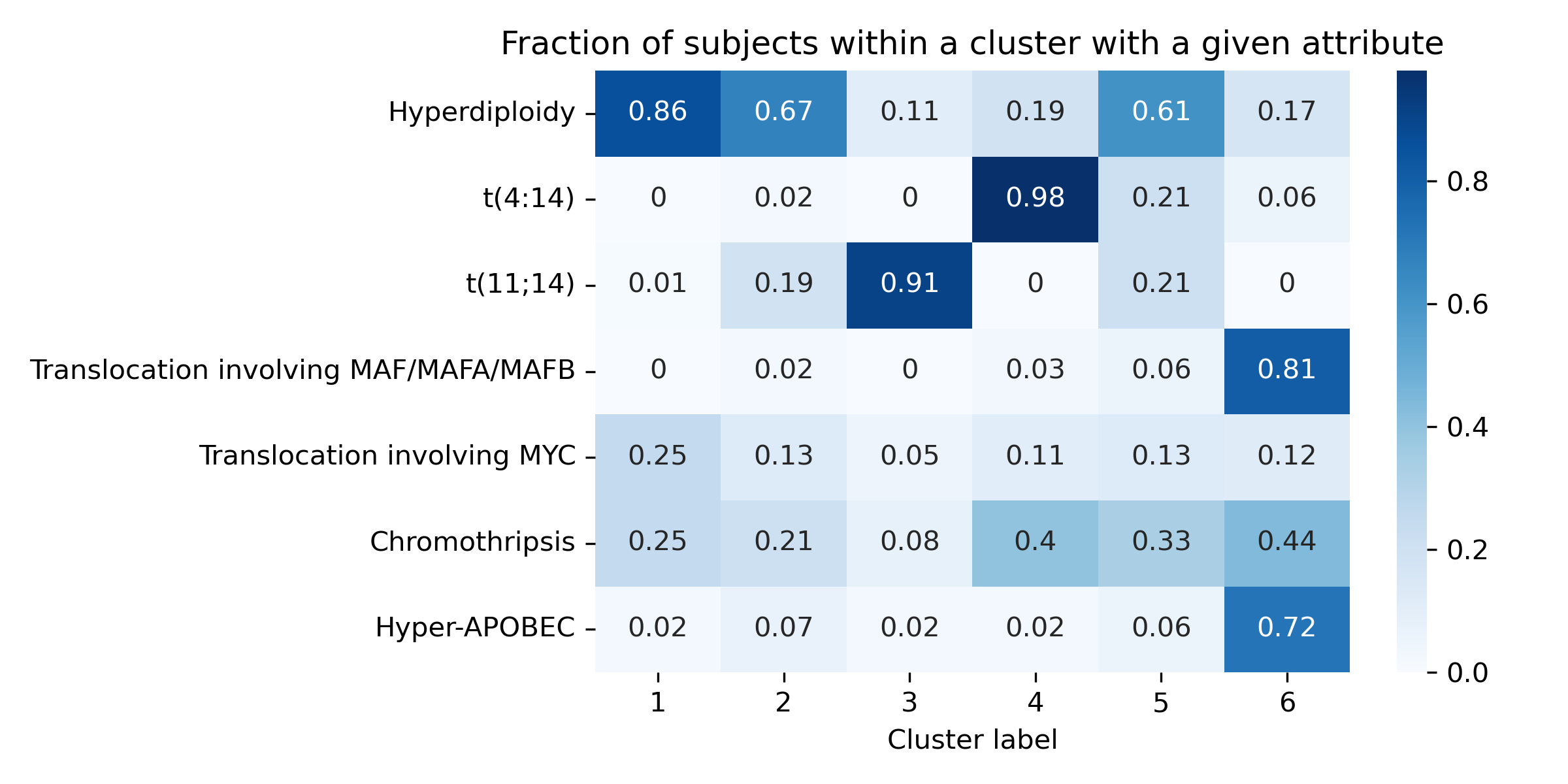


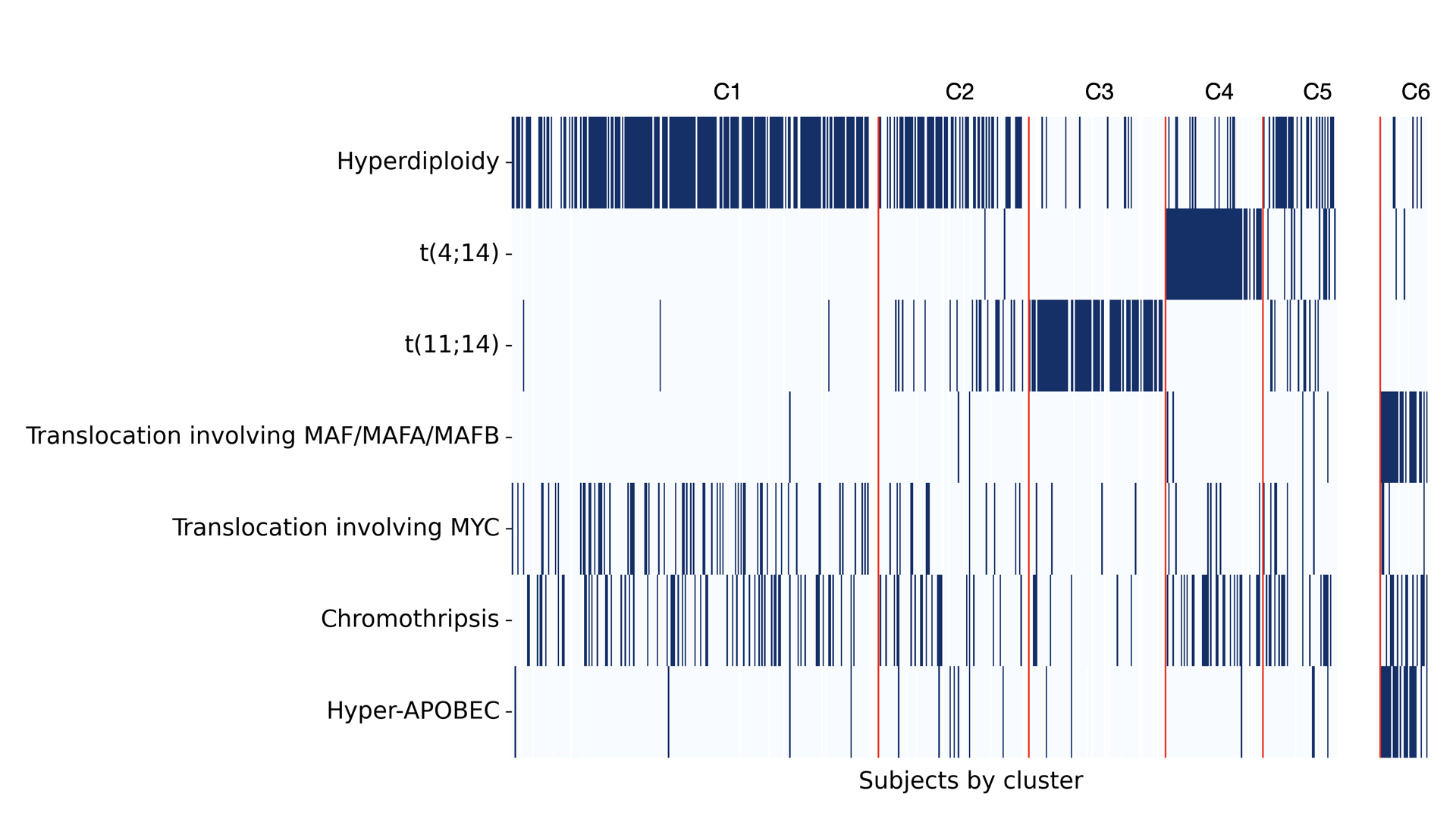


**Supplementary Figure 5. Scatter plot showing the relationship between copy number and RNA-seq expression values for RNF115.**


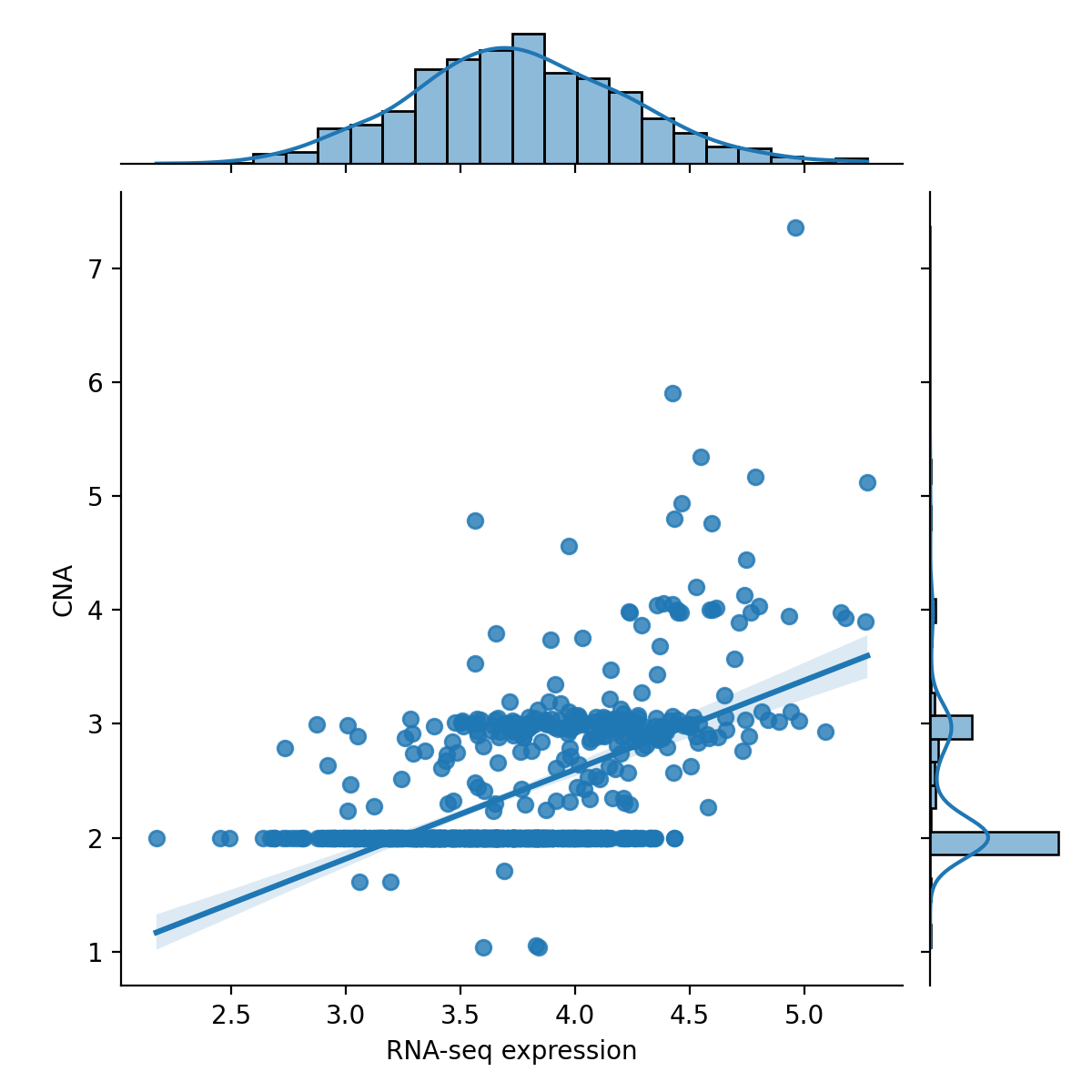


**Supplementary Figure 6. Near-neighbor analysis of gene-interactions for selected genes of significance in multiple myeloma biology based on copy number aberration.**

Each line or edge represents the interaction between a gene-pair in a network, comparing the median interactions observed in the high-risk group compared with those in the low-risk group. Blue edges indicate that the connections are more robust in the high-risk group, while orange edges are more fragile, risk being defined by the RNA-seq-based clustering analysis.

A B C


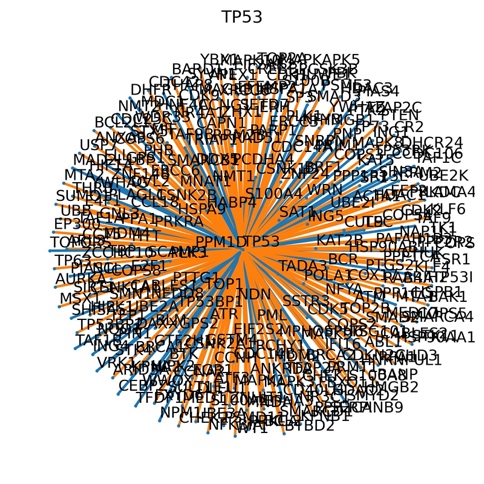

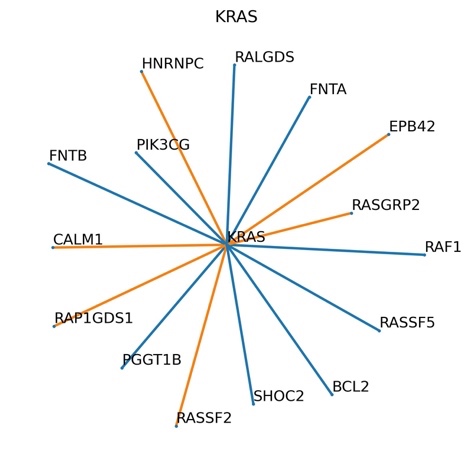

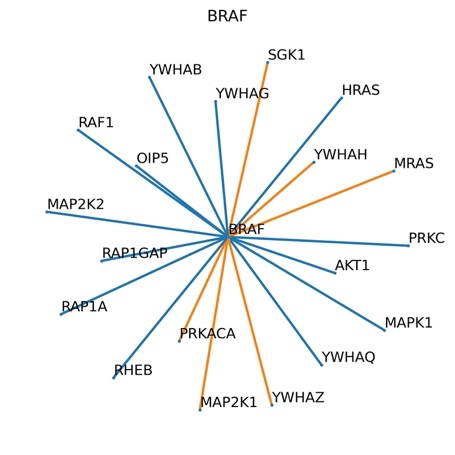


D E F


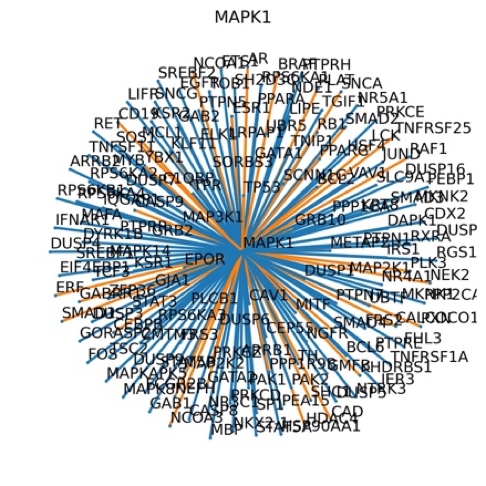

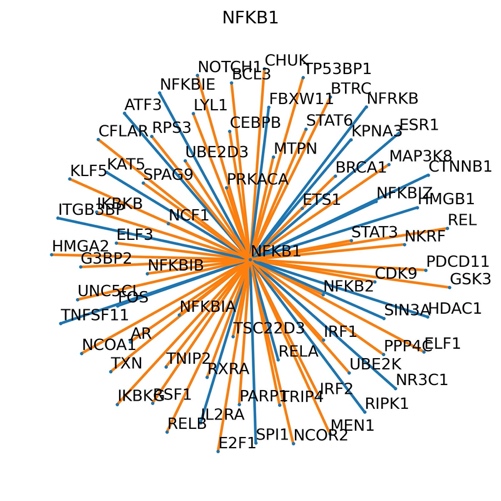

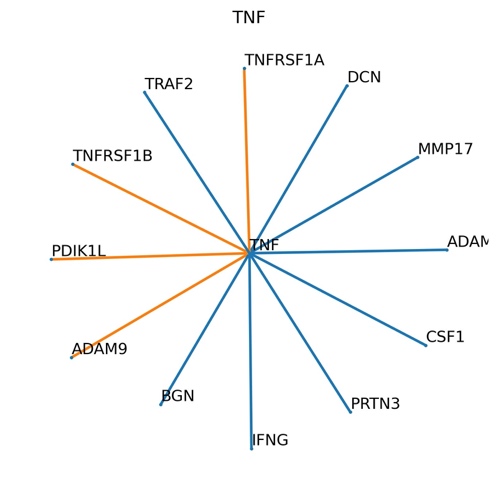


G H I


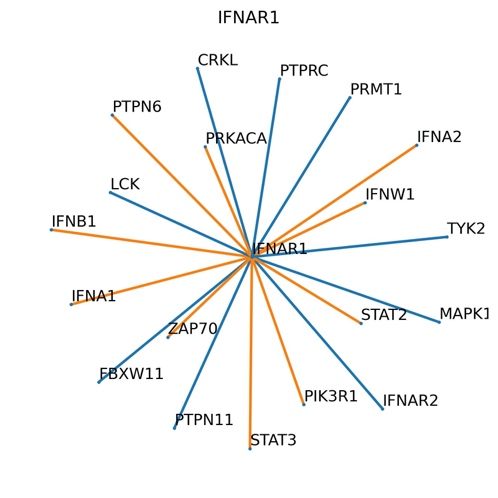

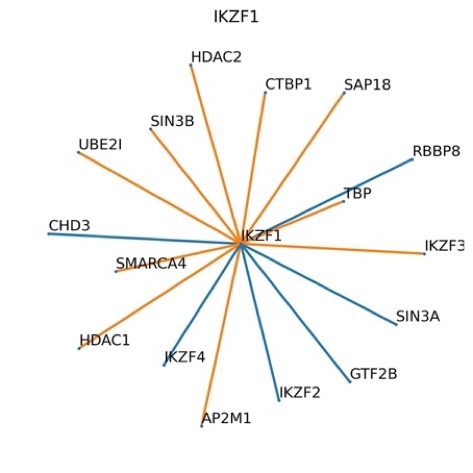

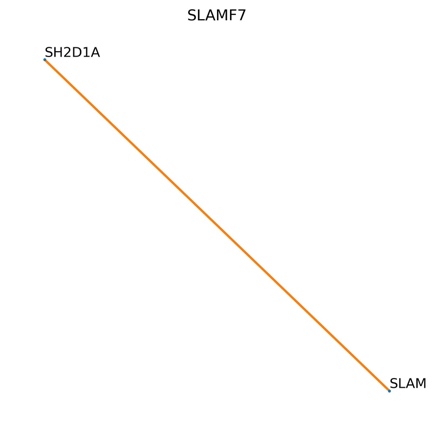


**Supplementary Figure 7. Near-neighbor analysis of gene-interactions for genes of prognostic significance in multiple myeloma biology based on copy number aberration.**

*PAM* presented as (A) 1-hop and (B) 2-hop network, *RNF115* presented as (C) 1-hop and (D) 2-hop network, and (E) *SNCAIP* 2-hop network. Each line or edge represents the interaction between a gene-pair in a network, comparing the median interactions observed in the high-risk group compared with those in the low-risk group. Blue edges indicate that the connections are more robust in the high-risk group, while orange edges are more fragile, risk being defined by the RNA-seq-based clustering analysis.

A B C


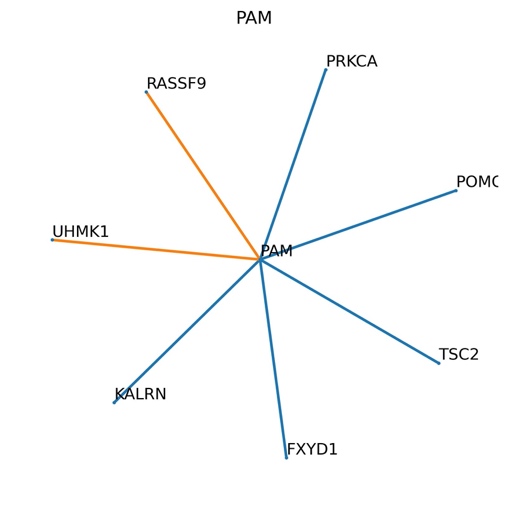

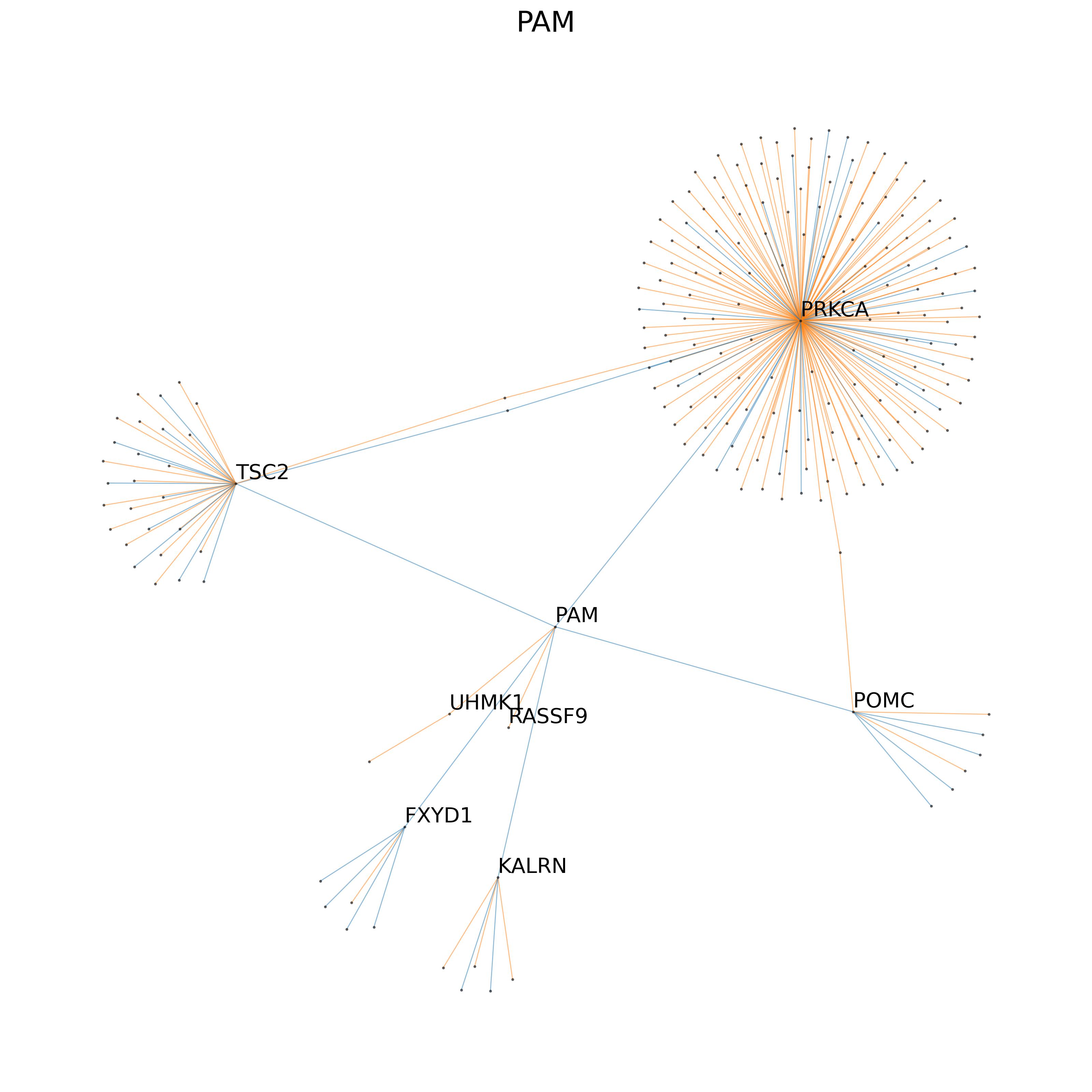

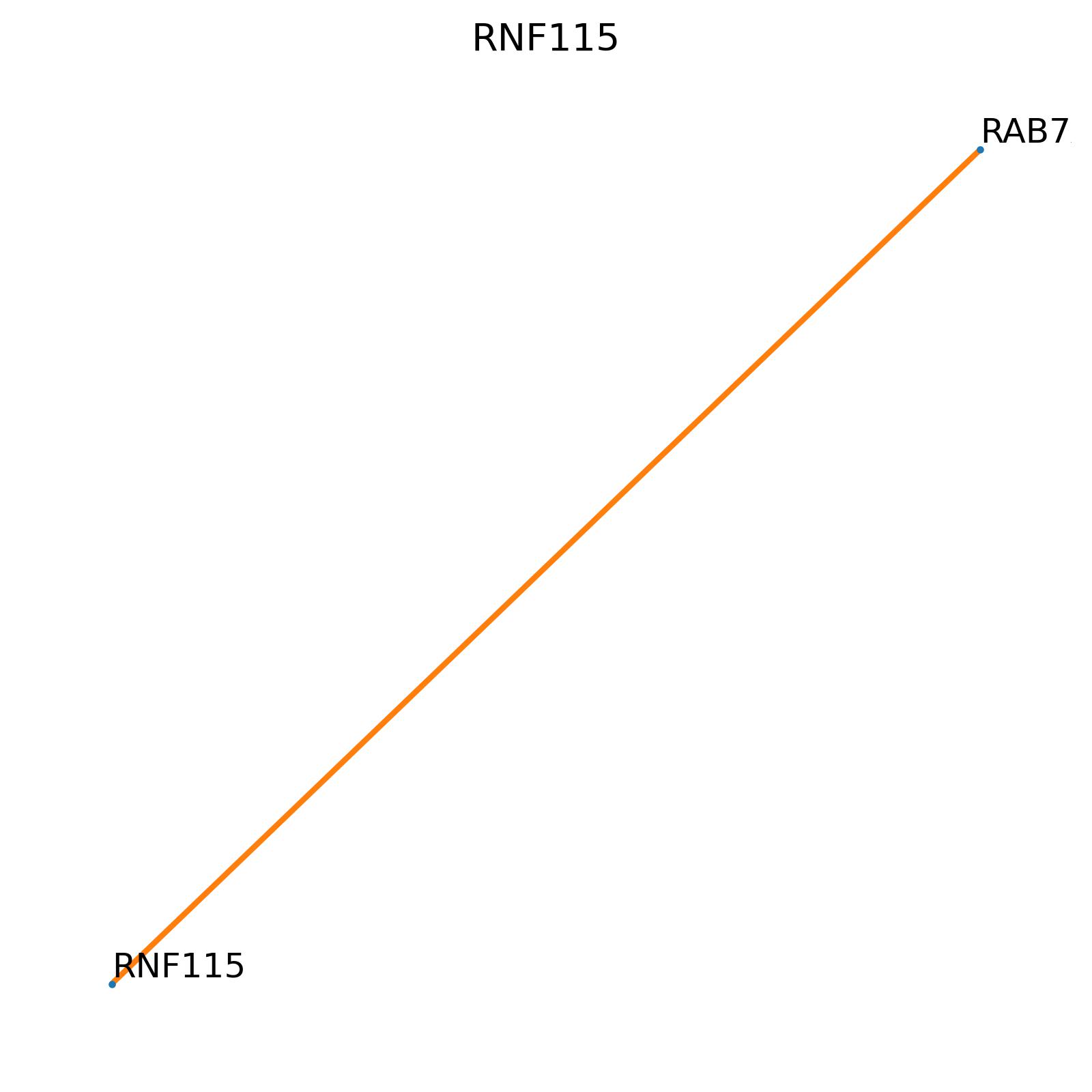


D E


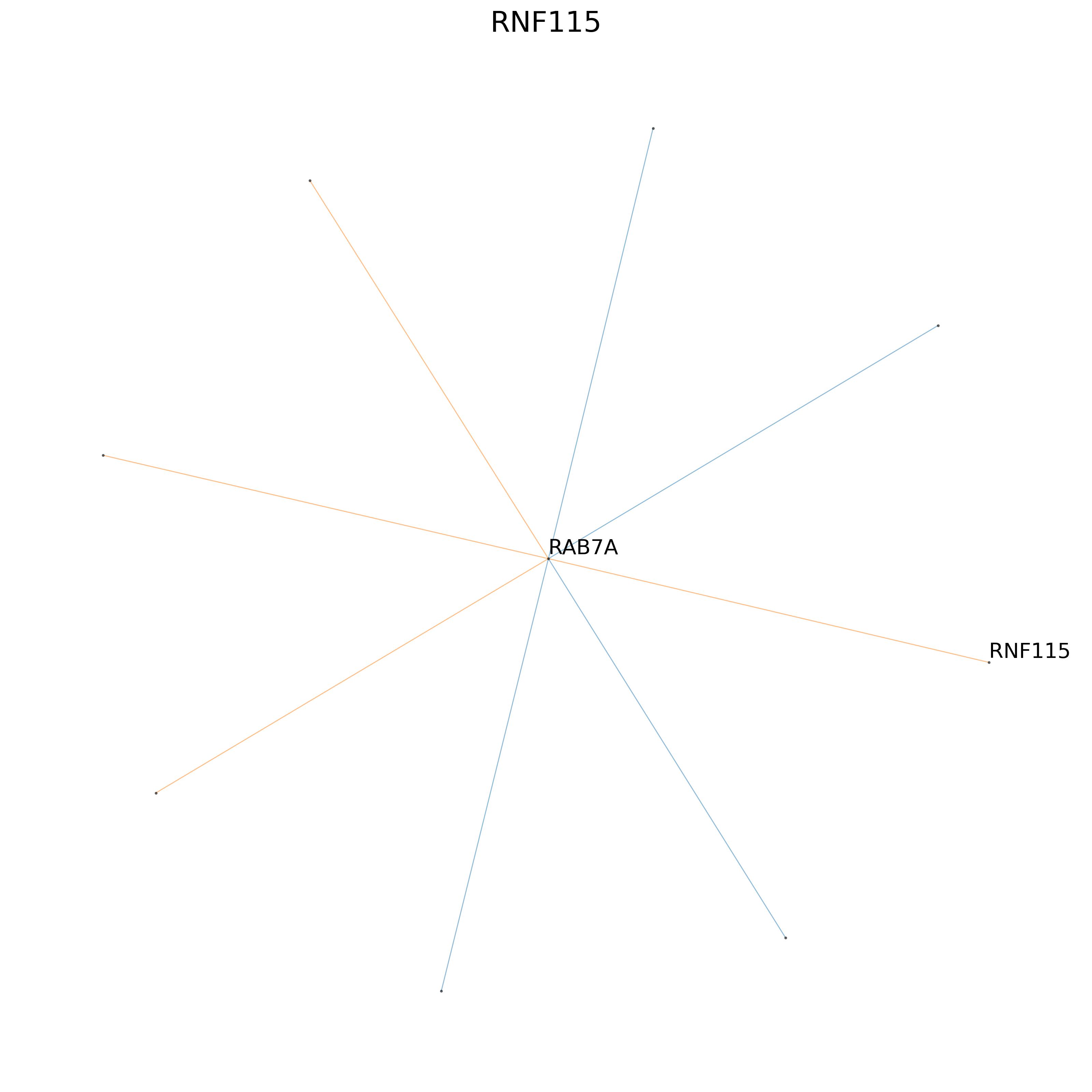

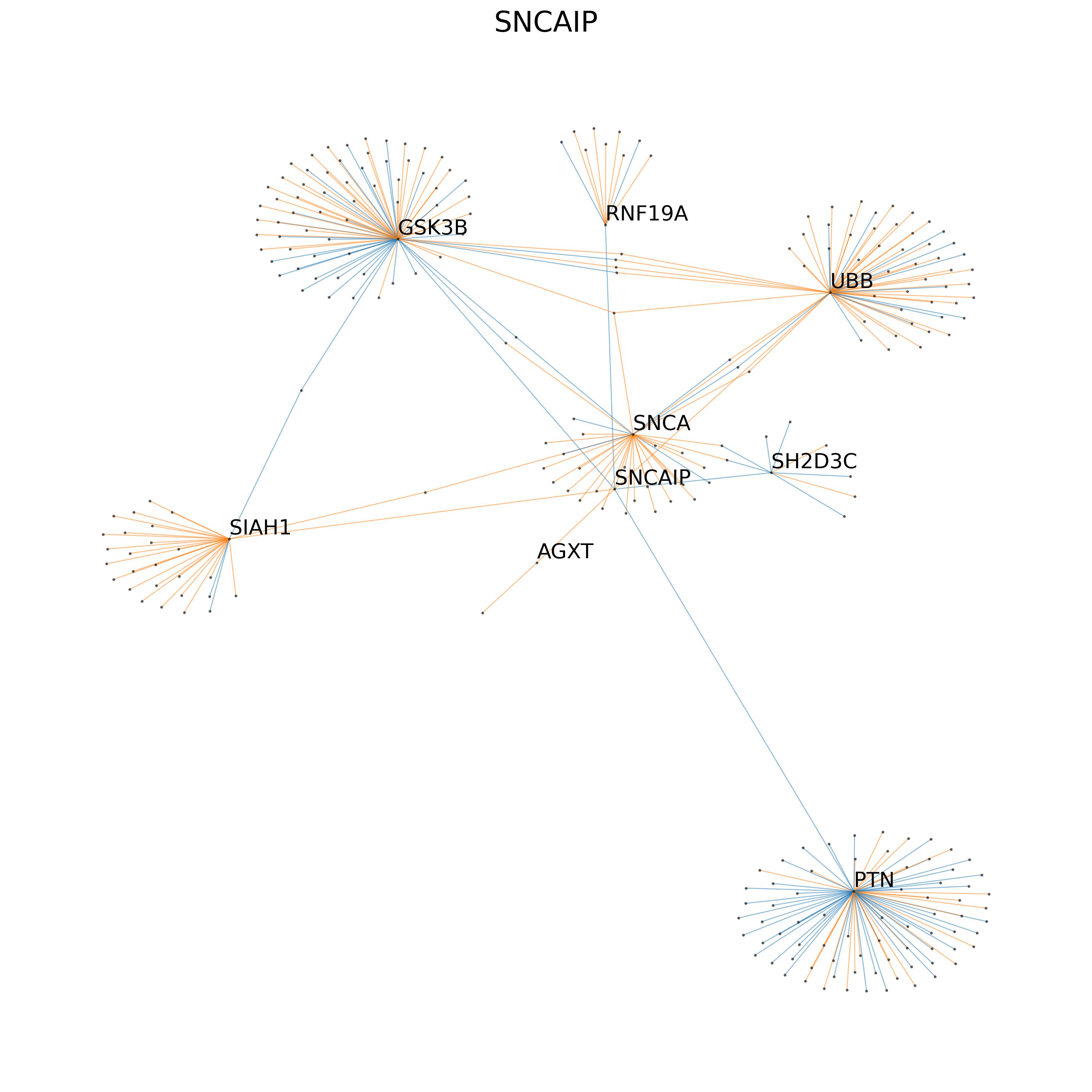


**Supplementary Figure 8.** Results of a multivariate cox proportional hazards (CPH) model for PFS. This model shows how the ORC-based definition of risk is relevant in the context of chromothripsis, APOBEC-mutational activity, gain/amp 1q and TP53 aberration. Left) Multivariate CPH for PFS covariates. Right) P-values associated with the multivariate CPH model.


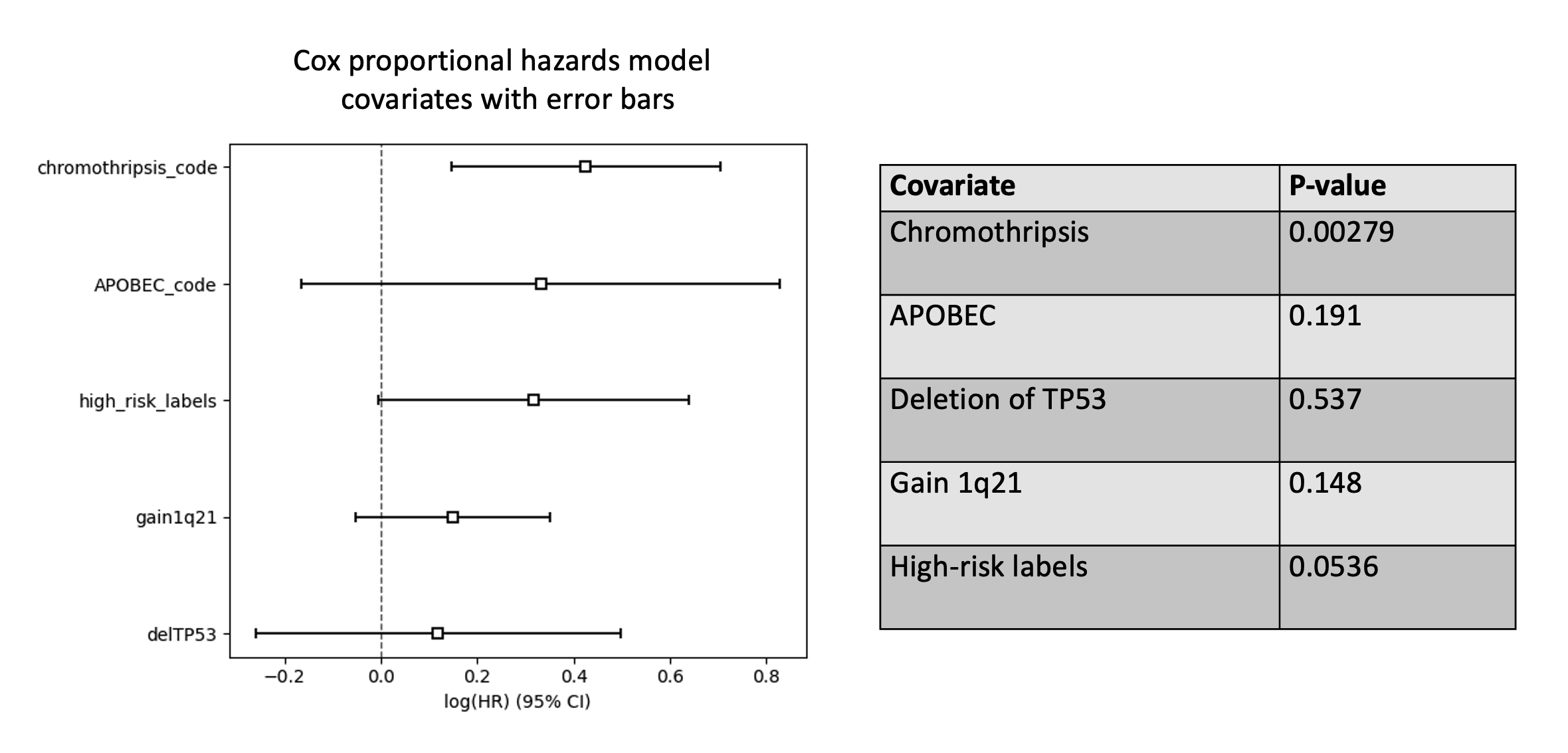


**Supplementary Table 1. Patient characteristics.**

| Variables | Patients (N=659) |
| --- | --- |
| Sex | Male: 393  Female: 266 |
| Age (mean ± SD) | 61.58 ± 13.09 years |
| ISS | Stage I: 229  Stage II: 232  Stage III: 179 |
| Treatment class | Bortezomib-based: 138  Carfilzomib-based: 38  IMIDs-based: 32  Combined bortezomib/IMIDs-based: 317  Combined IMIDs/carfilzomib-based: 110  Other: 24 |

**Supplementary Table 2. Patient clusters based on copy number aberration.**

| **Patient cluster** | **1** | **2** | **3** | **4** | **5** | **6** | **7** | **8** |
| --- | --- | --- | --- | --- | --- | --- | --- | --- |
| **Hyperdiploidy** | 145/152 | 13/65 | 2/36 | 83/85 | 3/80 | 34/36 | 7/52 | 26/37 |
| **t(4:14)** | 3/168 | 24/70 | 11/37 | 2/88 | 6/82 | 1/38 | 24/58 | 8/38 |
| **t(11;14)** | 6/168 | 5/70 | 17/37 | 2/88 | 65/82 | 1/38 | 13/58 | 1/38 |
| **Translocation involving *MAF/MAFA/MAFB*** | 3/168 | 2/70 | 6/37 | 1/88 | 4/82 | 1/38 | 15/58 | 2/38 |
| **Translocation involving *MYC*** | 32/168 | 11/70 | 5/37 | 24/88 | 3/82 | 6/38 | 11/58 | 4/38 |
| **Chromothripsis** | 48/168 | 11/70 | 8/37 | 21/88 | 12/82 | 7/38 | 25/58 | 15/38 |
| **gain1q21** | (0): 139; (1): 12; (2): 1 | (0): 41; (1): 21; (2): 3 | (0): 26; (1): 5; (2): 5 | (0): 43; (1): 40; (2): 2 | (0): 78; (1): 2 | (0): 24; (1): 12 | (0): 2; (1): 33; (2): 17 | (0): 18; (1): 17; (2): 2 |
| ***TP53* aberration** | (0): 113; (1): 16; (2): 7 | (0): 54; (1): 5 | (0): 28; (1): 1; (2): 3 | (0): 71; (1): 11 | (0): 56; (1): 7; (2): 5 | (0): 29 | (0): 43; (1): 5; (2): 1 | (0): 24; (1): 8; (2): 2 |
| **Hyper-APOBEC** | 6/167 | 3/70 | 8/36 | 1/88 | 5/81 | 3/38 | 11/58 | 3/38 |

**Key:** for gain 1q21, 0 = diploid, 1 = gain (3 copies), 2 = amplification (4 or more copies). For *TP53* aberration, 0 = diploid, 1 = either deletion or mutation, 2 = biallelic loss.

**Supplementary Table 3. Patient clusters based on RNA sequencing.**

| **Patient cluster** | **1** | **2** | **3** | **4** | **5** | **6** |
| --- | --- | --- | --- | --- | --- | --- |
| **Hyperdiploidy** | 197/229 | 62/93 | 9/82 | 12/63 | 28/46 | 5/30 |
| **t(4:14)** | 0/247 | 2/98 | 0/85 | 64/65 | 11/52 | 2/32 |
| **t(11;14)** | 3/247 | 19/98 | 77/85 | 0/65 | 11/52 | 0/32 |
| **Translocation involving *MAF/MAFA/MAFB*** | 1/247 | 2/98 | 0/85 | 2/65 | 3/52 | 26/32 |
| **Translocation involving *MYC*** | 61/247 | 13/98 | 4/85 | 7/65 | 7/52 | 4/32 |
| **Chromothripsis** | 62/247 | 21/98 | 7/85 | 26/65 | 17/52 | 14/32 |
| **gain1q21** | (0): 154; (1): 66; (2): 9 | (0): 70; (1): 21; (2): 2 | (0): 72; (1): 10 | (0): 33; (1): 18; (2): 12 | (0): 30; (1): 15; (2): 1 | (0): 12; (1): 12; (2): 6 |
| ***TP53* aberration** | (0): 178; (1): 24; (2): 5 | (0): 69; (1): 9; (2): 3 | (0): 60; (1): 5; (2): 4 | (0): 49; (1): 7; (2): 3 | (0): 40; (1): 4; (2): 1 | (0): 22; (1): 4; (2): 2 |
| **Hyper-APOBEC** | 4/247 | 7/97 | 2/83 | 1/65 | 3/52 | 23/32 |

**Key:** for gain 1q21, 0 = diploid, 1 = gain (3 copies), 2 = amplification (4 or more copies). For *TP53* aberration, 0 = diploid, 1 = either deletion or mutation, 2 = biallelic loss.

**Supplementary Table 4. 118 genes with significantly differential expression between high-risk and low-risk patient clusters.**

| Gene | FDR-BH corrected p value | log2FoldChange |
| --- | --- | --- |
| ACTB | 8.15E-09 | -3.9791242 |
| ADA | 6.49E-49 | -3.6033571 |
| ANAPC1 | 2.29E-51 | -3.6378753 |
| ANKS3 | 2.18E-34 | -3.8578852 |
| APBB2 | 7.39E-60 | 4.17615932 |
| ATP6AP1 | 4.47E-14 | -4.0398878 |
| ATXN7L3 | 4.02E-58 | -5.0704603 |
| BACH2 | 0 | -11.442387 |
| BCL9 | 2.20E-37 | -4.7917991 |
| BIN1 | 1.61E-12 | 3.95216124 |
| BRSK2 | 2.74E-104 | -6.6614015 |
| BUB1 | 3.01E-42 | -3.7545566 |
| C16orf74 | 3.61E-25 | -3.5439076 |
| C20orf27 | 5.64E-46 | -4.2841877 |
| CACNA1D | 8.33E-53 | -4.8883489 |
| CAPNS1 | 3.16E-23 | -4.3470666 |
| CBX4 | 8.72E-23 | -3.9309193 |
| CCL7 | 1.32E-70 | -4.7256429 |
| CCNO | 3.78E-30 | -4.9069896 |
| CD4 | 7.77E-75 | 4.71988701 |
| CDK18 | 9.11E-66 | -3.8441406 |
| CEACAM5 | 2.02E-31 | -3.6537205 |
| CHD3 | 1.80E-51 | -3.5925837 |
| CMPK1 | 3.26E-36 | -4.6679916 |
| COL4A3 | 1.06E-35 | -3.6036391 |
| COL4A5 | 4.72E-51 | 3.84907315 |
| COPS6 | 4.68E-163 | -6.6641661 |
| CUEDC1 | 1.21E-10 | -4.0609393 |
| CYP17A1 | 2.31E-67 | -3.5271311 |
| DBNDD2 | 7.51E-31 | -4.8378601 |
| DCAF8 | 1.03E-27 | -4.1124428 |
| DCTN4 | 1.17E-42 | -3.9372953 |
| DIS3L2 | 6.92E-30 | 3.89946868 |
| DOCK1 | 1.78E-56 | -4.9897879 |
| DYNC1LI1 | 5.12E-30 | -4.0480792 |
| EFCAB6 | 5.05E-25 | -3.6954477 |
| EIF1AD | 7.93E-125 | 6.37947139 |
| ERCC4 | 5.03E-09 | -4.3403268 |
| F2RL2 | 2.25E-65 | -6.3013585 |
| GDI1 | 9.03E-54 | -3.9927621 |
| GEMIN4 | 2.30E-34 | 3.93722446 |
| GHRH | 1.56E-108 | -7.817523 |
| GPSM3 | 2.17E-37 | -3.9933201 |
| GTF2F2 | 4.29E-49 | 6.74322358 |
| GTF2H5 | 1.75E-70 | -6.1578766 |
| HNRNPA1 | 9.63E-17 | -3.5015788 |
| IFNGR1 | 5.15E-80 | -5.2430709 |
| IL17RD | 2.26E-31 | 4.50350572 |
| IL2RA | 4.79E-28 | -6.0764612 |
| IMPA1 | 3.39E-23 | 3.5340342 |
| INCA1 | 5.84E-57 | -5.5860732 |
| INHBC | 3.97E-92 | -4.2723328 |
| JUN | 7.05E-24 | -4.3067353 |
| KCNJ2 | 2.61E-79 | -4.2186756 |
| KIF1B | 1.72E-52 | -3.6607465 |
| LATS1 | 1.11E-35 | 4.08705167 |
| LINGO1 | 1.26E-15 | -4.697893 |
| LRP5 | 1.04E-36 | 3.66044924 |
| LTBP4 | 2.65E-41 | -3.6075486 |
| MADCAM1 | 3.42E-85 | -4.0795823 |
| MAP3K6 | 1.77E-66 | -3.9558842 |
| MAVS | 2.70E-44 | -3.7527404 |
| MCM6 | 1.31E-21 | -3.5672485 |
| MED25 | 1.46E-10 | -4.1479156 |
| MEP1A | 1.31E-52 | -3.8316063 |
| MITF | 4.97E-83 | -3.5094859 |
| MLLT1 | 1.65E-38 | -8.7694144 |
| MLLT6 | 5.72E-34 | -5.7953935 |
| MUSK | 3.83E-31 | -3.6255295 |
| NCR3 | 4.59E-71 | -4.6162681 |
| NDN | 1.18E-31 | -4.1169187 |
| NFX1 | 2.19E-39 | -7.1611357 |
| NOL3 | 7.73E-74 | -5.5187276 |
| NOL8 | 1.98E-07 | -3.901438 |
| NOSTRIN | 3.51E-18 | -4.5085776 |
| NR2F1 | 1.47E-46 | 4.41516163 |
| NR5A1 | 7.16E-56 | -5.4338555 |
| PAK1 | 1.32E-47 | -5.1234588 |
| PAM | 6.59E-13 | -4.2158121 |
| PDPN | 1.14E-63 | -5.7685641 |
| PLIN3 | 3.89E-17 | -4.2807054 |
| PLK2 | 1.18E-28 | -4.4832599 |
| PMAIP1 | 1.57E-66 | 3.83360295 |
| PML | 5.49E-36 | -4.4926764 |
| POMP | 2.94E-12 | -3.6300316 |
| PPM1E | 2.33E-35 | -3.9880589 |
| PPP2R5E | 6.08E-14 | -3.7655002 |
| PPP3CC | 1.46E-34 | -4.3351609 |
| PRKG1 | 7.79E-50 | 4.41086621 |
| PTPRD | 3.00E-21 | -4.3537645 |
| RAB3GAP2 | 8.87E-50 | -4.199373 |
| RAP1GAP | 5.90E-52 | -7.1112586 |
| RBMXL2 | 2.20E-34 | -3.608101 |
| REXO4 | 4.04E-28 | -4.9149512 |
| RNF115 | 1.36E-30 | -5.2538633 |
| S100Z | 1.92E-32 | -5.6034288 |
| SAT1 | 8.61E-14 | -3.9051086 |
| SCG5 | 1.76E-58 | -3.7836285 |
| SEC24B | 4.41E-79 | -5.5362465 |
| SENP6 | 1.14E-44 | -4.4886736 |
| SERPINA10 | 1.34E-26 | -3.6655945 |
| SLC16A2 | 3.99E-21 | -3.7403086 |
| SLC37A1 | 4.49E-75 | -5.1199062 |
| SMARCA2 | 4.00E-64 | -3.6407725 |
| SNCAIP | 4.97E-21 | -3.5787914 |
| SNRNP200 | 1.65E-08 | -4.5538716 |
| SNX22 | 1.78E-24 | 3.76276335 |
| SOX2 | 8.15E-63 | 4.53096046 |
| SPRR2A | 8.60E-72 | -4.2878226 |
| ST13 | 1.79E-39 | -5.3182038 |
| SUMF2 | 1.27E-50 | -3.541777 |
| SYN1 | 2.80E-13 | -5.0970774 |
| TEP1 | 1.98E-38 | -5.4549138 |
| TP53RK | 8.31E-41 | 3.53965215 |
| TPP1 | 1.98E-68 | -4.498507 |
| ULBP3 | 1.02E-18 | -4.6317657 |
| VSNL1 | 1.94E-27 | -5.3289795 |
| WEE1 | 1.50E-28 | 3.80049792 |

**Supplementary Table 5. Average RNA-seq value by RNA clusters**

|  | BUB1 | MCM6 | NOSTRIN | PAM | RNF115 | SNCAIP | SPRR2A | WEE1 |
| --- | --- | --- | --- | --- | --- | --- | --- | --- |
| RNA-C1 | 3.007856 | 3.59871 | 0.299373 | 6.177293 | 3.665073 | 0.169891 | 0.206924 | 1.977061 |
| RNA-C2 | 3.255012 | 3.596 | 0.454455 | 5.908047 | 3.577369 | 0.238021 | 0.239468 | 2.511178 |
| RNA-C3 | 3.721532 | 3.954352 | 0.269624 | 5.859292 | 3.845161 | 0.219357 | 0.007925 | 3.481427 |
| RNA-C4 | 3.844088 | 3.835831 | 0.228146 | 5.650652 | 4.07145 | 0.150612 | 1.109568 | 2.610448 |
| RNA-C5 | 2.721099 | 3.009289 | 0.297748 | 5.816373 | 3.766678 | 0.124581 | 0.206298 | 3.092492 |
| RNA-C6 | 3.709875 | 4.114429 | 0.254172 | 5.647126 | 4.077781 | 0.641875 | 0.744075 | 3.449972 |

**Supplementary methods**

***Curvature-based edge ranking analysis***

Using ORC, the strength of connections originating from a gene can be summarized by computing the scalar curvature for a given node. Scalar curvature is the summation of edge curvature values originating in the node (gene) of interest and is formally defined below.

[
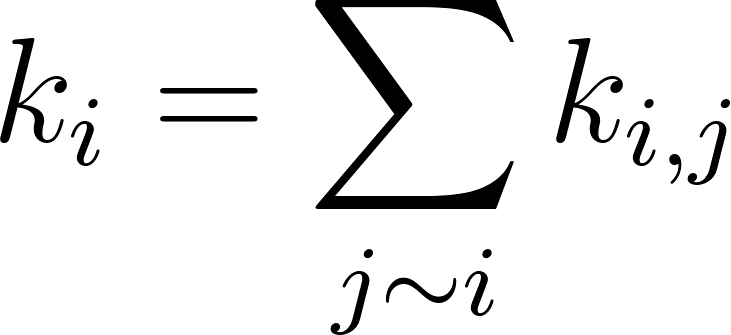
](https://www.codecogs.com/eqnedit.php?latex=%20k_i%20%3D%20%5Csum_%7Bj%20%5Csim%20i%7D%20k_%7Bi%2C%20j%7D%20#0)

ORC for RNA-Seq and CNA data was computed separately for each patient. To consolidate the geometric information induced by the nodal weights (RNA-Seq and CNA), we computed the pure topological curvature (wherein all the weights were set to be 1) and subtracted that from each of the original curvature values for all patients. Once computed, gene pairs were then ranked based on the computed value which we call the strength of curvature.
